# Prevalence and morphometry of sternal and xiphoid foramen: a meta-analysis on 16,666 subjects

**DOI:** 10.1007/s00276-023-03116-9

**Published:** 2023-03-15

**Authors:** Patrycja Pasieka, Paweł Melchior Pasieka, Alexander Komosa, Agnieszka Barnowska, Jakub Pękala, Konrad Malinowski, Krzysztof Tomaszewski

**Affiliations:** 1 International Evidence-Based Anatomy Working Group, Krakow, Poland; 2 Department of Anatomy, Jagiellonian University Medical College, Kopernika 12, 31-034 Kraków, Poland; 3 Faculty of Medicine and Health Sciences, Andrzej Frycz Modrzewski Kraḱów University, Kraków, Poland

**Keywords:** Sternal body, Xiphoid process, Perforated sternum, Sternal biopsy, Thoracic anatomy

## Abstract

**Purpose:**

Sternal foramen is a perforation of the sternum that can be a source of misdiagnosis during radiographic imaging or life-threatening perforations during bone marrow sampling. The aim of this study was to conduct a meta-analysis on the prevalence, morphometrics, and location of foramen in the sternal body and xiphoid process, describe morphometric features of this phenomenon, and thus verify its clinical importance. Moreover, our secondary outcome was to compare effectiveness of various imaging methods in diagnosis of the sternal or xiphoid foramen.

**Methods:**

A comprehensive search was conducted on major scientific databases to identify studies containing relevant information. Data on foramen’s prevalence, location, morphometrics, and accompanying findings were extracted and pooled into a meta-analysis using MetaXL 5.0.

**Results:**

Thirty-five studies (*n* = 16,666 subjects) were included. The overall pooled prevalence of a foramen in the sternal body and/or a xiphoid process was 8.9% (95% CI 6.5–11.7) and it equaled 6.5% (95% CI 5.6–7.6) for sternal body alone and 2.9% (95% CI 0.5–6.9) for the xiphoid process. The foramen was more prevalent in males than in females (12.2% vs. 6.8%). The prevalence of sternal foramen was higher in South American [13.9% (95% CI 11.2–16.9)] and African [13.6% (95% CI 9.7–18.0)] studies compared to North American [6.2% (95% CI 5.0–7.5)] and European populations [8.6% (95% CI 3.1–16.3)]. Mean transverse and vertical diameter of foramen equaled 4.7 mm (95% CI 3.8–5.5), and 5.6 mm (95% CI 4.2–6.9), respectively.

**Conclusion:**

Our analysis proves that the sternal foramina are structures of significant prevalence and size. Any physician should keep them in mind when performing punctures in this area.

## Introduction

**Fig. 1:**
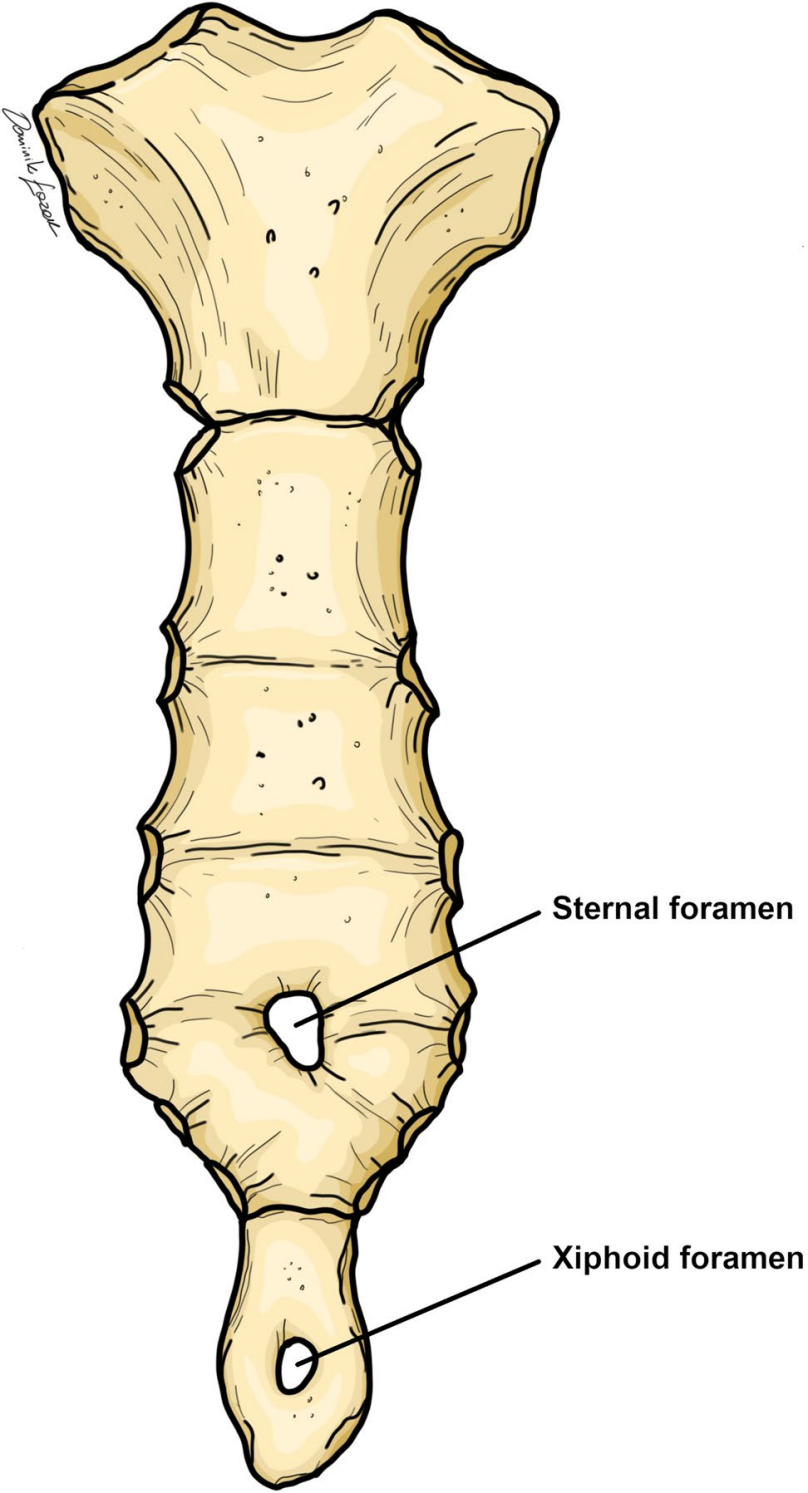
Exemplary presentation of sternal foramen

Sternal foramen is a developmental defect of the sternum that results in complete perforation in its manubrium, body, and/or xiphoid process. This anatomic variant may present singly or multiple times anywhere along the sternum (Fig. 1). The embryological origins of sternal foramen are thought to arise from the imperfect union of the sternal bars, the embryological structural origin of the sternum [3, 25, 28]. The foramen was first observed in 1649 and first described in 1707 [11]. Studies on the prevalence of foramen in the sternum have been intensively investigated in both cadaveric and radiographic studies [X-ray, Multidetector computed tomography (MDCT), Computed tomography (CT), and Magnetic resonance imaging (MRI)]. In the studies collected for the purpose of this meta-analysis, the prevalence of a sternal or xiphoid foramen in individual research reports ranged from 0.2% [40] to as much as 57.8% [42]. In clinical practice, sternal foramina are usually discovered incidentally or as a cause of complications during medical procedures [9]. Cross-sectional imaging modalities (CT, MDCT, and MRI) are usually required to accurately assess the structure of the sternum to determine presence of foramen in a clinical setting.

The awareness of sternal foramen has clinical significance for bone marrow biopsies, radiographic imaging, and acupuncture as the sternum lies in proximity of vital structures, such as pericardium and the lung. In bone marrow biopsies and acupunctures, sternal foramen may be penetrated, leading to damage to underlying structures of which damage to the pericardium has resulted in cardiac tamponades and even death [11]. For radiologists, it is possible for sternal foramen to be potentially confused for different pathologies, such as fractures, gun shots, and lytic bone lesions [15]. Therefore, awareness of the existence of foramen in the sternum during training of these professions is crucial.

The aim of this study was to provide the most comprehensive and evidence-based assessment to date of the prevalence of sternal foramen, its anatomical features including morphometric data, and associations with sternal clefts, and thus verify its clinical importance. Moreover, our secondary outcome was to compare effectiveness of various imaging methods in diagnosis of the sternal or xiphoid foramen. To accomplish this task, we conducted a meta-analysis, including all studies containing extractable data that have been published on foramen in the sternum, in all languages without any time restrictions.

## Materials and methods

### Search strategy

An extensive search of the major electronic databases (PubMed, Embase, ScienceDirect, CNKI, SciELO, BIOSIS, Web of Science, Core Collection, Current Content Connect, Korean Journal Database, Russian Citation Index, and Google Scholar) up to June 2022 was conducted to identify all studies which reported relevant information on foramen in the sternal body and xiphoid process foramen. No date limits or language restrictions were applied. The search terms used for this meta-analysis were: sternal foramen OR xiphoid foramen OR perforated sternum.

The authors further performed a search through the references of all included articles to identify additional studies potentially eligible for inclusion in the meta-analysis. The authors strictly followed Preferred Reporting Items for Systematic Reviews and Meta-Analyses (PRISMA) guidelines while performing this study.

### Eligibility assessment

Eligibility assessment was performed by two independent reviewers. All peer-reviewed studies reporting extractable data on the prevalence and/or anatomical characteristics of sternal and xiphoid foramen were included into the meta-analysis. The following exclusion criteria were employed: (1) case studies, reviews, letters to editors, essays, and conference abstracts; (2) studies containing irrelevant, incomplete, or contradictory data; (3) studies containing animal and fetal studies. There was no need to contact any study authors to resolve issues during the meta-analysis. No language or time restrictions were applied. In case of any disagreements during eligibility assessment, all decisions were made by a consensus among all of the authors. The Anatomical Quality Assessment (AQUA) Tool was utilized to evaluate the risk of bias in enrolled studies.

### Data extraction

Two independent reviewers conducted data extraction. Data on the prevalence, location, number (single vs multiple) of sternal and xiphoid foramina as well as gender of subjects, nationality of subjects, co-existence of sternal clefts, and methods of gathering data (autopsy, CT, MRI) were extracted. Whenever possible, more specific information regarding the location and size of the foramen in the sternum and xiphoid process was given. More specific information could be the distance in millimeters of sternum and xiphoid process foramen in relation to the superior border of manubrium and/or inferior border of xiphoid process, the intercostal space or vertical and horizontal size of the foramen. In case of any discrepancies regarding data within a study, a decision was reached by unanimous agreement between the reviewers.

### Statistical analysis

Statistical analysis of the pooled prevalence of the sternal foramen was conducted using MetaXL 5.3 by EpiGear International Pty Ltd (Wilston, Queensland, Australia). The morphometrics parameters were pooled using Comprehensive Meta-Analysis version 3.0 by Biostat (Englewood, New Jersey, USA). A random effects model was applied for all analyses. To assess the heterogeneity of included studies, chi^2^ test and *I*^2^ statistic were utilized. Significant heterogeneity among studies in chi^2^ test was defined as Cochran’s Q *p*-value < 0.10. For the *I*^2^ statistic, interpretation was performed based on the following intervals: 0–30%—“might not be important”, 30–60%—“might indicate moderate heterogeneity”, 50–90%—“may indicate substantial heterogeneity”, 75–100%—“may represent considerable heterogeneity”.

Single-categorical pooled prevalence was calculated. The studies that gathered data only on the part of the sternum (e.g., xiphoid process or manubrium) were excluded from general pooled prevalence calculation.

To probe the sources of heterogeneity, subgroup analysis by the imaging modality, sex, location of foramen (specific rib), and geographical region (continent and country) was conducted, when appropriate. The analysis of foramen location and number of foramina (single vs. multiple) was also performed for subject populations known to have a foramen in the sternum. Furthermore, a sensitivity analysis inclusive of studies with sample size equal or greater than 200 subjects, was performed when appropriate to further investigate the source heterogeneity. To probe for statistically significant differences between groups, confidence intervals were compared, and if they overlapped, the differences between groups were considered as statistically insignificant.

## Results

### Study identification

**Fig. 2:**
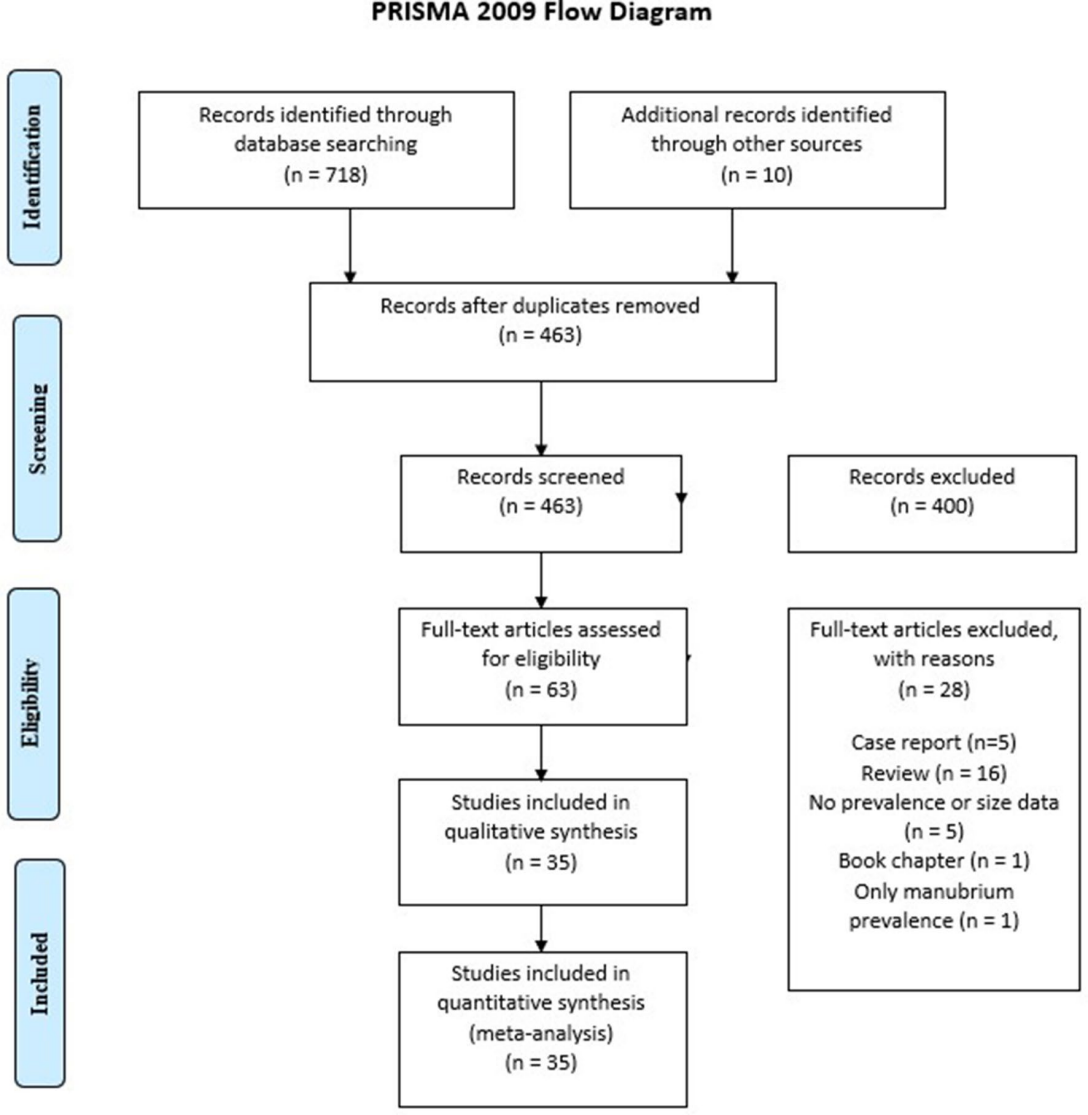
PRISMA flow chart of the meta-analysis

The process of study identification is presented in Fig. 2. A total of 718 articles were identified in an extensive search of the electronic databases. An additional 10 articles were identified by searching through the references of the articles identified in the electronic database search. A total of 63 full text articles were assessed by authors for potential eligibility. After excluding articles for containing exclusion criteria as listed above as well as duplicates, 35 articles were included into the meta-analysis [1, 2, 4–10, 12, 14, 16–20, 22–24, 26, 27, 29, 31, 33–39, 41–45].

### Characteristics of included studies

**Fig. 3:**
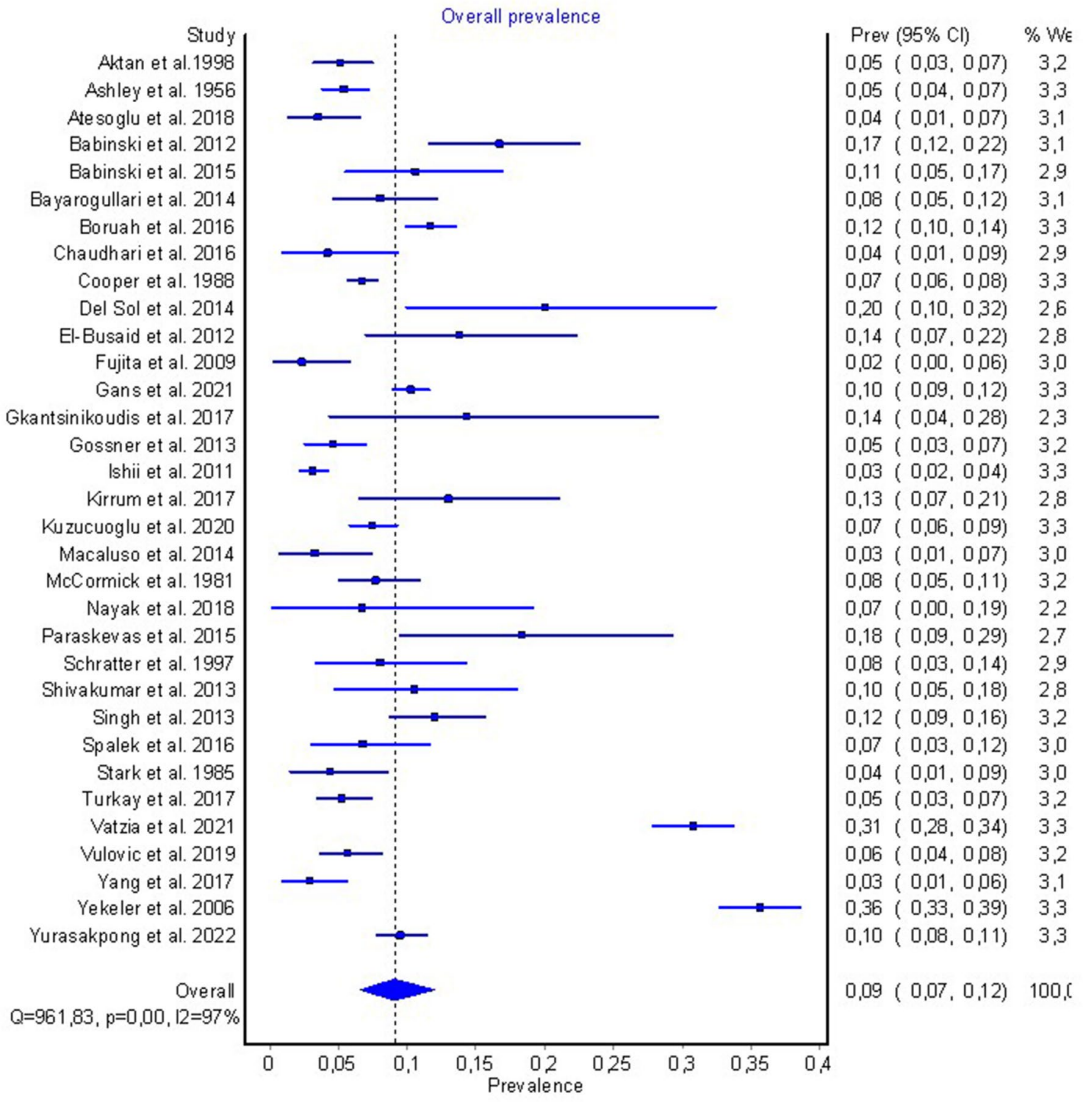
Forest plot of the overall pooled prevalence of sternal/xiphoid foramen

The characteristics of included studies are presented in Table 1. 35 studies (*n* = 16,666) were included in this meta-analysis (Fig. 3). A total of 33 studies (*n* = 15,223 subjects) were included into the quantitative analysis. (Fig. 3), while 2 studies (*n* = 1443) were analyzed separately in Table 3, as they contained data only on foramen in the xiphoid process. The breakout of the studies with respect to study sample, imaging modality, and geographic origin has been included in Table 1. Several studies have been split into two subgroups for sub-analyses as they included separate study samples belonging to different modalities and/or geographic groups. One study [40] assessed foramen’s prevalence only in the sternal manubrium and thus was not pooled in any analysis.

**Table 1:** Characteristics of included studies

First author and year	Country	Type of study	Number of patients	Sternal/xiphoid foramen prevalence (%)
Akin et al. 2011 [1]	Turkey	MDCT	500	43.2
Aktan et al. 1998 [2] (a)	Turkey	Cadaveric	62	3.2
Aktan et al. 1998 [2] (b)	Turkey	HRCT	350	5.4
Ashley et al. 1956 [4] (a)	UK (African sample)	Xray	98	13.3
Ashley et al. 1956 [4] (b)	UK	Xray	573	4.0
Atesoglu et al. 2018 [5]	Turkey	MDCT	200	3.5
Babinski et al. 2015 [6]	Brazil	MDCT	114	10.5
Babinski et al. 2012 [7]	Brazil	cadaveric	180	16.7
Bayarogullari et al. 2014 [8]	Turkey	MDCT	200	8.0
Boruah et al. 2016 [9]	India	MDCT	1180	11.6
Chaudhari et al. 2016 [10]	India	Cadaveric	96	4.2
Cooper et al. 1988 [12]	USA	Xray	2016	6.7
Del Sol et al. 2014 [14]	Chile	Cadaveric	50	20.0
El-Busaid et al. 2012 [16]	Kenya	Cadaveric	80	13.8
Fujita et al. 2009 [17]	Japan	CT	129	2.3
Gans et al. 2021 (a) [18]	Bolivia	CT	1334	13.0
Gans et al. 2021 (b) [18]	USA	CT	703	5.0
Gkantsinikoudis et al. 2017 [19]	Greece	Cadaveric	35	14.3
Gossner et al. 2013 [20]	Germany	CT	352	4.5
Ishii et al. 2011 [22]	Japan	MDCT	1053	3.1
Kirrum et al. 2017 [23]	Uganda	Cadaveric	85	12.9
Kuzucuoglu et al. 2020 [24]	Turkey	CT	912	7.5
Macaluso et al. 2014 [26]	Spain	X-ray	122	3.3
McCormick et al. 1981 [27]	USA	X-ray	324	7.7
Nayak et al. 2018 [29]	India	Cadaveric	30	6.7
Paraskevas et al. 2015 [31]	Greece	Cadaveric	60	18.3
Schratter et al. 1997 [33]	Austria	CT	100	8.0
Shivakumar et al. 2013 [34]	India	Cadaveric	86	10.5
Singh et al. 2013 [35]	India	Cadaveric	343	12.0
Spalek et al. 2016 [36]	Poland	CT	134	6.7
Stark et al. 1985 [37]	USA	CT	138	4.3
Turkay et al. 2017 [38]	Turkey	MDCT	500	5.2
Vatzia2021 [39]	Greece	MDCT	950	30.7
Verna et al. 2015 [40]	France	CT	502	0.2
Vulovic et al. 2019 [41]	Serbia	MDCT	422	5.7
Xie et al. 2013a [42]	Korea, China	MDCT	902	57.8
Xie et al. 2013b [42]	Korea, China	Cadaveric	41	56.1
Yang et al. 2017 [43]	China	CT	212	2.8
Yekeler et al. 2006 [44]	Turkey	MDCT	1000	35.6
Yurasakpong2022 [45]	Thailand	CT	1000	9.5

*MDCT* multidetector computed tomography, *HRCT* high−resolution computed tomography, *CT* computed tomography

The included articles showed a wide geographical distribution with studies performed in Asia, Europe, North America, South America, and Africa.

### Prevalence of sternal and xiphoid foramen

A total of 33 studies (*n* = 15,223 subjects) reported data on the prevalence of a sternal or xiphoid foramen, geographic location, and imaging modality (Table 2). The overall pooled prevalence of a sternal and/or xiphoid foramen in the study population was 8.9% (95% CI 6.5–11.7). A sensitivity analysis was also performed, including only studies with a sample size equal to or greater than 200. The pooled prevalence of sternal and/or xiphoid foramen in this group was 8.1% (95% CI 5.1–11.7). In a subgroup analysis by study type, there was no statistically significant difference between the radiographic study types (MDCT, CT, and X-ray), but there was a statistically significant difference between cadaveric studies and the radiographic studies, with the exception of MDCT.

**Table 2:** Total pooled prevalence of sternal and xiphoid foramen in population

Category	Number of studies (number of subjects)	Pooled prevalence: % (95% CI)	*I*^2^: (95% CI)	*p*-value
Overall	33 (15,223)	8.9 (6.5–11.7)	96.5 (95.9–97.1)	< 0.001
Males	20 (5871)	12.2 (8.5–16.4)	94.7 (93.0–96.0)	< 0.001
Females	19 (3750)	6.8 (4.8–9.0)	80.6 (70.5–87.2)	< 0.001
Sensitivity	20 (13,753)	8.1 (5.1–11.7)	98.0 (97.6–98.4)	< 0.001
Sternal body	31 (13,057)	6.5 (5.6–7.6)	79.5 (71.9–85.1)	< 0.001
Xiphoid process	33 (14,436)	2.9 (0.5–6.9)	99.1 (99.0–99.2)	< 0.001
Cadaveric	12 (1431)	11.4 (8.7–14.5)	61.3 (27.5–79.4)	< 0.001
MDCT	9 (5619)	10.5 (3.7–19.8)	98.9 (98.6–99.1)	< 0.001
CT	10 (5014)	6.3 (4.3–8.7)	88.3 (80.6–93.0)	< 0.001
X-ray	4 (3133)	6.2 (5.1–7.5)	33.0 (0.0–76.2)	0.215
Africa	3 (263)	13.6 (9.7–18.0)	0.0 (0.0–0.0)	0.988
Asia	15 (6853)	7.5 (3.8–12.3)	97.5 (96.8–98.1)	< 0.001
Europe	10 (3248)	8.6 (3.1–16.3)	97.5 (96.5–98.2)	< 0.001
North America	4 (3181)	6.2 (5.0–7.5)	34.1(0.0–76.8)	0.208
South America	4 (1678)	13.9 (11.2–16.9)	32.2 (0.0–75.8)	0.219

*MDCT* multidetector computed tomography, *CT* computed tomography, *CI* confidence interval

The geographical subgroup analysis showed that the only statistically significant difference in sternal foramen prevalence was that South American and African populations had a higher prevalence of sternal foramen than North American and European populations (Table 2).

The presence of a foramen in the sternum was more common among males than females, with a pooled prevalence of 12.2% (95% CI 8.5–16.4) and 6.8% (95% CI 4.8–9.0) respectively (Table 2). Among the general population with studies evaluating at the entire sternum and giving the information on location of foramen (33 studies, *n* = 14,436 subjects), the pooled prevalence of a xiphoid foramen was 2.9% (95% CI 0.5–6.9). In studies that examined only the xiphoid process (3 studies, *n* = 1443 subjects), the prevalence of a foramen was significantly higher at 51.9% (95%CI 40.0–63.6) (Table 3).

**Table 3:** Prevalence of xiphoid foramen (only xiphoid process examined)

Category	Number of studies (number of subjects)	Pooled prevalence: % (95% CI)	*I*^2^: (95% CI)	*p*-value
Overall	3 (1443)	51.9 (40.0–63.6)	92.8 (82.1–96.1)	< 0.001
Analysis without Akin et al. 2011 [1]	2 (943)	57.7 (54.5–60.8)	0.0 (0.0–0.0)	0.819
Analysis without Xie et al. 2013a [42]	2 (541)	47.4 (35.6–59.3)	60.0 (0.0–90.7)	0.114
Analysis without Xie et al. 2013b [42]	2 (1402)	50.6 (36.2–65.0)	96.4 (90.0–98.7)	< 0.001

*CI* confidence interval

### The pooled prevalence of a single foramen and multiple foramen in study populations

The overall pooled prevalence of a single foramen in the sternum (20 studies, *n* = 6724 subjects) was 9.2% (95% CI 6.3–12.5) (Table 4). The overall pooled prevalence of multiple foramen in the sternum was 0.5% (95% CI 0.1–1.0) (Table 4).

**Table 4:** Pooled prevalence of single and multiple foramen in population

Category	Number of studies (number of subjects)	Pooled prevalence: % (95% CI)	*I*^2^: (95% CI)	*p*-value
Single foramen				
Overall	20 (6724)	9.2 (6.3–12.5)	94.1 (92.1–95.6)	< 0.001
Asia	10 (4259)	8.3 (6.8–9.9)	59.1 (17.7–79.6)	0.009
Europe	6 (1091)	5.8 (4.2–7.6)	41.3 (0.0–75.3)	0.073
Multiple foramen				
Overall	20 (6724)	0.5 (0.1–1.0)	77.8 (66.2–85.4)	< 0.001

*CI* confidence interval

### Location of a foramen in the sternal body and xiphoid process in subject populations with a foramen

Table 5 gives the pooled prevalence of the location of the foramen, in subjects with a foramen (31 studies, *n* = 1466 subjects), as well as the location of the foramen with respect to the numbered intercostal segment. Table 5 gives additional statistics on sex, imaging, and geographic prevalences of sternal and xiphoid foramen respectively.

**Table 5:** Location of the foramen (subjects with foramen)

Category	Number of studies (number of foramina)	Pooled prevalence: % (95% CI)	*I*^2^: (95% CI)	*p*-value
At 4th intercostal segment and 4th costal pit	4 (187)	22.1 (0.0–56.0)	92.6 (84.3–96.5)	< 0.001
At 5th intercostal segment and 5th costal pit	6 (211)	62.9 (33.3–88.4)	90.9 (83.0–95.1)	< 0.001
Sternal body				
Overall	31 (1466)	89.4 (74.3–98.7)	97.9 (97.5–98.2)	< 0.001
Males	9 (357)	82.4 (46.8–100.0)	97.1 (95.9–98.0)	< 0.001
Females	9 (127)	94.5 (42.9–97.0)	87.9 (79.7–92.7)	< 0.001
Cadaveric	12 (172)	86.5 (70.3–97.4)	82.6 (71.6–89.4)	< 0.001
MDCT	9 (903)	86.0 (51.6–100.0)	99.1 (98.9–99.3)	< 0.001
Asia	13 (1066)	82.4 (53.5–99.6)	98.7 (98.3–98.9)	< 0.001
Europe	11 (431)	85.3 (51.9–100.0)	97.3 (96.3–98.0)	< 0.001
Xiphoid process				
Overall	33 (1441)	10.5 (1.8–24.0)	97.4 (96.9–97.8)	< 0.001
Males	5 (295)	36.8 (1.3–82.3)	95.9 (93.0–97.7)	< 0.001
Females	5 (103)	50.0 (17.8–82.2)	85.3 (67.6–93.4)	< 0.001
Cadaveric	12 (172)	12.7 (2.4–28.1)	81.6 (69.7–88.9)	< 0.001
MDCT	9 (903)	14.5 (0.0–41.5)	98.7 (98.2–99.0)	< 0.001
Asia	11 (741)	15.4 (0.6–40.1)	97.3 (96.4–98.0)	< 0.001

*CI* confidence interval

Table 6 provides information on the location of the sternal foramen, as measured from the superior border of the manubrium and from the inferior tip of the xiphoid process in millimeters, respectively.

**Table 6:** Location of foramen—mean distance (mm)

Measurement points	Number of studies (number of subjects)	Mean distance (mm) (95% CI)	*I*^2^
From the superior border of manubrium to the foramen	3 (186)	119.9 (119.3–120.4)	88.0
From the foramen to the inferior tip of xiphoid process	2 (49)	70.0 (69.2–70.7)	98.2

*CI* confidence interval

### Size of the foramen

In the sub-analysis of foramen’s size (5 studies, *n* = 218 subjects), the mean transverse size of the foramen was 4.7 mm (95% CI 3.8–5.5), and the vertical size was 5.6 mm (95% CI 4.2–6.9) (Table 7).

**Table 7:** Size of the sternal foramen

Category	Number of studies (number of subjects)	Mean size (mm) (95% CI)	*I*^2^
Transverse	5 (218)	4.7 (3.8–5.5)	81.5
Vertical	5 (218)	5.6 (4.2–6.9)	87.9

*CI* confidence interval

### Single and multiple foramen among subjects with foramina

In study subjects with foramina, the pooled prevalence of single foramen in the sternum (20 studies, *n* = 869 subjects) was 95.0% (95% CI 90.3–98.2) (Table 8). For multiple foramina, the prevalence was 4.4% (95% CI 2.1–7.6) (Table 8).

**Table 8:** Number of subjects with single/multiple foramen among subjects with foramina

Category	Number of studies (number of subjects with foramen)	Pooled prevalence: % (95% CI)	*I*^2^: (95% CI)	*p*-value
Single foramen				
Whole sternum	20 (869)	95.0 (90.3–98.2)	76.9 (64.6–84.9)	< 0.001
Sternal body	11 (182)	98.9 (96.7–100)	0.0 (0.0–28.5)	0.851
Xiphoid process	10 (780)	85.6 (75.2–93.6)	84.0 (72.2–90.8)	< 0.001
Multiple foramen				
Whole sternum	20 (869)	4.4 (2.1–7.6)	56.7 (28.6–73.7)	0.001
Sternal body	11 (182)	1.1 (0.0–3.3)	0.0 (0.0–28.5)	0.851
Xiphoid process	11 (816)	12.6 (5.6–21.6)	83.0 (71.0–90.0)	< 0.001

*CI* confidence interval

### Prevalence of sternal clefts in subjects with foramen in the sternal body

Table 9 provides pooled prevalence of sternal clefts in subjects with foramen in the sternal body. The pooled prevalence of sternal cleft in subjects with foramen in the sternal body was 9.6% (CI 2.2–20.7).

**Table 9:** Prevalence of sternal cleft in patients with foramen in sternal body

Category	Number of studies (number of subjects)	Pooled prevalence: % (95% CI)	*I*^2^: (95% CI)	*p*-value
Overall	5 (228)	9.6 (2.2–20.7)	71.7 (28.7–88.8)	0.007

*CI* confidence interval

### Risk of bias assessment

The results of an AQUA-Tool analysis have been summarized in (Table 10). The significant portion of analyzed studies professed “high” risk of bias in Domain 3, either because of inadequate efforts to reduce inter-/intra-observer variability or vague description of applied methods. Additionally, several studies scored “high” risk of bias in Domain 1 due to insufficient size of the sample or lack of information regarding demographic and baseline data of the subjects.

**Table 10:** Results of AQUA-tool analysis

Study	Risk of bias				
	Objective(s) and study characteristics	Study design	Methodology characterization	Descriptive anatomy	Reporting of results
Akin et al. 2011 [1]	Low	Low	Low	Low	Low
Aktan et al. 1998 [2]	Unclear	Low	High	Unclear	Low
Ashley et al. 1956 [4]	Unclear	Unclear	High	Low	Low
Atesoglu et al. 2018 [5]	Low	Low	Low	Low	Low
Babinski et al. 2012 [7]	Low	Low	High	Low	Unclear
Babinski et al. 2015 [6]	Low	Low	Low	Low	Low
Bayarogullari et al. 2014 [8]	Low	Low	High	Low	Low
Boruah et al. 2016 [9]	Low	Low	Low	Low	Low
El-busaid et al. 2012 [16]	Low	Low	High	Low	Low
Chaudhari et al. 2016 [10]	High	Low	High	Low	Low
Cooper et al. 1988 [12]	Low	Low	High	High	Low
Delsol et al. 2014 [14]	Unclear	Low	High	Low	Low
Fujita et al. 2009 [17]	High	Low	High	Low	Low
Gans et al. 2021 [18]	Low	Low	Low	Low	Low
Gkantsinikoudis et al. 2017 [19]	High	Low	High	Low	Low
Gossner et al. 2013 [20]	Low	Low	High	Low	Low
Ishii et al. 2011 [22]	High	Low	High	Low	Low
Kirrum et al. 2017 [23]	Low	Low	Low	Low	Low
Kuzucuoglu et al. 2020 [24]	Low	Low	High	Low	Low
Macaluso et al. 2014 [26]	Low	Low	High	Low	Low
Nayak et al. 2018 [29]	High	Low	High	Low	Low
Paraskevas et al. 2015 [31]	Low	Low	High	Low	Low
Schratter et al. 1997 [33]	Low	Low	High	High	Low
Shivakumar et al. 2013 [34]	High	Unclear	High	High	Low
Singh et al. 2013 [35]	Low	Low	High	Low	Low
Spalek et al. 2016 [36]	Unclear	Low	High	Low	Low
Stark et al. 1985 [37]	High	Unclear	High	Unclear	Low
Turkay et al. 2017 [38]	Low	Low	High	Low	Low
Vatzia et al. 2021 [39]	Low	Low	Low	Low	Low
Verna et al. 2015 [40]	Low	Low	High	Low	Low
Vulovic et al. 2019 [41]	Low	Low	Low	Low	Low
Xie et al. 2013 [42]	Low	Low	Low	Low	Low
Yang et al. 2017 [43]	Low	Low	Low	Low	Low
Yekeler et al. 2006 [44]	Low	Low	Low	Low	Low
Yurasakpong et al. 2022 [45]	Low	Low	Low	Low	Low

## Discussion

This study aimed to collect and summarize published evidence on the prevalence and morphometry of sternal foramen into a comprehensive meta-analysis. This was the first study reporting pooled prevalence of foramina in the sternum, pooled prevalence of the location of single and multiple foramen in the general population, as well as population with existing foramen in the sternum.

Our meta-analysis, which was based on more than 16,000 subjects, found that the presence of foramen in the sternum is relatively common. The pooled prevalence of a foramen in the sternal body and/or xiphoid process was 8.9%.

It has been suggested that due to the frequency of sternal foramina, they are of clinical significance to healthcare providers, such as radiologists or thoracic surgeons. For healthcare providers, the presence of a sternal foramen is significant in cases where it is necessary to acquire bone marrow samples from the sternum. A needle used in a bone marrow biopsy can penetrate through the foramen and damage the underlying structures. According to a study of anatomical structures underneath the sternum, the pericardium is adjacent to a sternal foramen in 11–20% of cases [11], and the right ventricle is adjacent to more than 99% of sternums [30]. In literatures, there are at least 14 incidents of cardiac tamponade (of which 8 were fatal) that could be identified as a complication of sternal punctures [6].

In addition to cardiac damage during bone marrow biopsy, it is also possible for acupuncturists to cause damage to underlying structures via a foramen in the sternum. In 1995, a Norwegian woman died of a cardiac tamponade after an acupuncturist punctured the anterior wall of the right ventricle [21]. Babinski with colleagues attempted trans-thoracic puncture through sternal foramen on 16 cadavers. In all cases, the right ventricle was damaged by the puncture [7]. The area of penetration in this cadaver study corresponded to Shangzong acupuncture point (CV 17) in 62.5% of cases, and the Zhongting acupuncture point (CV 16) in 38.5% of cases [13]. Two other structures susceptible to damage by penetration of a sternal foramen are the aorta and the lungs.

The challenge involved with procedures and acupunctures done in the region of the sternum is that sternal foramen are not usually identifiable by physical exam due to the presence of dense connective tissue overlying the foramen. Therefore, it is even more important for anyone conducting penetrations of the sternum to be aware of the possibility of penetrating sternal foramen.

With respect to radiology, it is important that radiologists are aware of the presence of sternal foramen, so that those anatomical structures are not mistaken for evidence of traumatic injury (for example, a bullet wound), or a lytic bone lesion [2, 15, 32]. With respect to diagnosis of a sternal foramen, it may not be detectable via bone scintigraphy. According to Atesoglu et al., Multidetector computed tomography (MDCT) imaging is considered more useful in wholly revealing sternal anatomy including sternal foramen [5].

Thus, because of the possibility of traumatic injury to underlying structures and misdiagnosis of pathologies in radiological investigations, we recommend that radiologists analyzing the region of the sternum, and acupuncturists as well as medical professionals undertaking invasive procedures in the area of sternum should be made aware of the existence of sternal foramen in their training.

In a sensitivity analysis including studies with more than 200 subjects, the total pooled prevalence was 8.1%. In studies that analyzed only the xiphoid foramen, the calculated pooled prevalence in these studies was 51.9%, significantly higher than the 2.9% found in general population studies that analyzed the entire sternum. It is not clear why the prevalence of xiphoid foramen is so high in this study population. One possible explanation is that in the process of excluding “sternal body only” articles from xiphoid process foramen prevalence analysis, it was not always clear whether the xiphoid process was properly examined; therefore, the prevalence could be inaccurate. The number of studies that focused only on the xiphoid process was very small (2 studies with 3 distinct samples), leaving this analysis vulnerable to potential bias in the studies. We believe that conducting more studies focusing exclusively on xiphoid processes might explain this discrepancy in reported prevalences.

A geographical subgroup analysis found that South Americans and African populations had the highest prevalence of sternal foramen. European and North American populations had the lowest prevalence rates of sternal foramen. There was no statistical difference between each pairing. Asian populations were not distinguishable from any group. The wide geographical variability suggests that the prevalence of a foramen in the sternal body might be influenced by genetics.

With respect to gender differences in sternal foramen prevalence, prevalence was higher in men compared to women. This could indicate that there are differences in the embryological development of foramen in the sternum, between the sexes. However, it is worth noticing that no statistically significant difference between sexes was found with respect to the prevalence of foramen in the xiphoid process.

The pooled prevalence of single foramen in the sternum in subject populations was significantly higher compared to the prevalence of multiple foramina. Therefore, even if one foramen is identified, although the risk of encountering another foramen is low, it is still necessary to keep in mind the possibility of additional foramen somewhere in the sternum.

When identifying the location of a foramen in the sternum, it is much more likely that the foramen will be present in the sternal body compared to the xiphoid process; therefore, more effort should be made to examine the sternal body for potential foramina. Some studies included in our analysis underlined the high prevalence of the foramina in the 5th intercostal segment and 5th costal pit, as well as the 4th intercostal segment and 4th costal pit.

### Imaging and foramen in the sternum

For the detection of sternal and xiphoid foramen in the general population, our analysis revealed that there is a statistically significant improvement in the detection of sternal foramen in cadaveric studies, versus CT and X-ray imaging modalities. There is, however, no statistically significant difference between MDCT, CT, and X-ray studies, nor is there a statistically significant difference between cadaveric and MDCT studies. This is likely due to the fact that in autopsies, it is possible to view small foramen that possibly could not be visualized reliably in MDCT, X-ray, and CT. Although there was no statistically significant difference between MDCT, CT, and X-ray, it is suggested that healthcare providers looking to ascertain the presence of a sternal foramen should use MDCT imaging [5].

## Limitations

The main limitation of our meta-analysis was the high heterogeneity among the included studies. Additionally, not all studies reported extractable data on the exact location of the foramen and number of foramina. Although the predominance of studies hailing from Asia (15 studies, *n* = 6853), Europe (10 studies, *n* = 3248), and North America (4 studies, *n* = 3181) compared to a relative lack of studies from Africa (3 studies, *n* = 263), South America (4 studies, *n* = 1678), and no studies from Oceania may have impacted the overall pooled prevalence rates, the authors conducted a geographical subgroups analysis finding that statistically significant differences existed only between South American/African and North American/European populations. Lastly, various methods (cadaveric, CT, and radiographs) utilized in studies may have slightly skewed the overall pooled prevalence. However, to reduce this effect, the authors conducted separate statistical analysis by study type detecting no significant statistical differences between imaging modalities.

## Conclusions

Foramen in the sternum is highly prevalent in the general population, being present in almost 10% of cases included in the meta-analysis. Awareness of the presence of sternal foramen is important during the planning of invasive procedures, such as bone marrow aspiration and acupuncture therapy. Additionally, sternal foramina are important to consider during diagnosis of suspected pathologies in radiology. Radiologists should be made aware of this anatomical variation during training so as not to make incorrect diagnosis. This meta-analysis could not clearly prove the superiority of using MDCT over other imaging modalities. However, given the limitations of this study, we believe that further large-scale and well-designed studies are needed to ascertain which imaging modality is the most sensitive. Despite the lack of certainty, based on the results of this meta-analysis, we would still recommend the use of MDCT based on authors’ observations in analyzed papers and how it was the only method comparable with cadaveric examination.

## Data Availability

All study data have been presented in the manuscript.
